# Resolving the heading‐direction ambiguity in vertical‐beam radar observations of migrating insects

**DOI:** 10.1002/ece3.5184

**Published:** 2019-05-03

**Authors:** Zhenhua Hao, Vincent Alistair Drake, John R. Taylor

**Affiliations:** ^1^ School of Science The University of New South Wales Canberra Australian Capital Territory Australia; ^2^ School of Biology Lund University Lund Sweden; ^3^ Institute for Applied Ecology University of Canberra Canberra Australian Capital Territory Australia

**Keywords:** Australian plague locust, heading direction, insect migration, orientation, radar entomology, selection method

## Abstract

1. Each year, massive numbers of insects fly across the continents at heights of hundreds of meters, carried by the wind, bringing both environmental benefits and serious economic and social costs. To investigate the insects' flight behavior and their response to winds, entomological radar has proved to be a particularly valuable tool; however, its observations of insect orientation are ambiguous with regard to the head/tail direction, and this greatly hinders interpretation of the migrants' flight behavior.
2. We have developed two related methods of using wind data to resolve the head/tail ambiguity, and we have compared their outputs with those from simply assigning the heading direction to be that which is closer to the track direction. We applied all three methods to observations of Australian plague locust migrations made with an insect monitoring radar.
3. For the study dataset, some of the headings selected by the simpler method are shown to be clearly incorrect. The two new methods generally agree and reveal a significantly different, and presumably more accurate, relationship of heading direction to track direction. However, use of these methods leads to quite a large proportion of the sample being lost because the wind values, which derive from a regional‐scale numerical model, are shown to be incompatible with the radar observations. This exploratory study has moreover demonstrated that locusts are frequently oriented at a large angle to their track and that quite often their movement is at least slightly tailfirst.
4. Both new methods appear to be a significant improvement on the simpler method. As well as providing an accurate representation of migratory flight behavior, they allow occasions when the model wind values are unreliable to be eliminated from the data sample.

## 1 INTRODUCTION

Migratory behavior in insects has evolved as an adaptation to environmental variability across space and through time (Drake & Gatehouse, 1995). Throughout the year, massive numbers of insects migrate across the globe. These migrations may lead to invasions of growing crops and consequent economic losses (Drake & Reynolds, 2012, pp 312–346; Koul, Cuperus, & Elliott, 2008), but also to huge seasonal exchanges of biomass and nutrients (Hu et al., 2016). Insect migration patterns and strategies have evolved differently among insect species, but the vehicle of movement is usually the wind. However, recent studies on large nocturnal migrants have shown that they are not undiscriminating passengers on the wind, but appear to optimize their migration trajectories by selecting favorable heights (Wood et al., 2006) or flying with a crosswind heading direction (or *orientation*—we will use these two terms interchangeably) to optimize their flight trajectories (Chapman et al., 2010). Medium‐sized (10–70 mg) nocturnal insect migrants are predicted to orient toward the right of the downwind direction in the northern hemisphere as they respond to turbulence cues resulting from the change of wind direction with altitude due to the effect of surface friction (the “Ekman spiral”; Reynolds, Reynolds, Smith, & Chapman, 2010). On the other hand, a larger moth, the silver Y (*Autographa gamma*; ~150 mg), was shown to have seasonally changing preferred migration directions, to compensate for wind drift, and to initiate migratory flight predominantly under tailwind conditions in order to gain ground speed and be carried toward their destination (Chapman et al., 2010). They fly with a significantly higher degree of drift compared to nocturnal songbird migrants, thus exhibiting lower precision in orientation (Chapman et al., 2015). These findings and recent investigations of the orientation mechanisms used by high‐flying insects in darkness (Dreyer et al., 2018) are leading to improved understanding of flight behavior, and how these behaviors guide insects of a variety of species to regions with seasonally favorable climates and habitat resources (Chapman et al., 2012).

Radar observation has been a major tool for developing our understanding of insect migrations (Chapman, Drake, & Reynolds, 2011; Drake & Reynolds, 2012, pp 74–99). Recently, several studies have achieved insights by combining radar observations with detailed meteorological information from high‐resolution atmospheric models (Boulanger et al., 2017; Reynolds, Chapman, & Drake, 2017; Westbrook & Eyster, 2017). The radar automatically records the insects' track directions and speeds of travel relative to the ground (the track vector ***T***) and also their body alignments (Drake, 2002). The insect track direction, the direction toward which the insect is traveling, is measured as an azimuthal angle between 0° and 360° (clockwise from north). However, insect body alignment is an axial direction between 0° and 180°, also clockwise from north, that is, the head and tail of the insects cannot be distinguished so there is a 180° ambiguity in the orientation. Some scanning radars do provide additional clues suggesting how the ambiguity should be resolved, but these are not clear‐cut or easily interpreted (Drake, 1983; Melnikov, Istok, & Westbrook, 2015).

**Figure 1:**
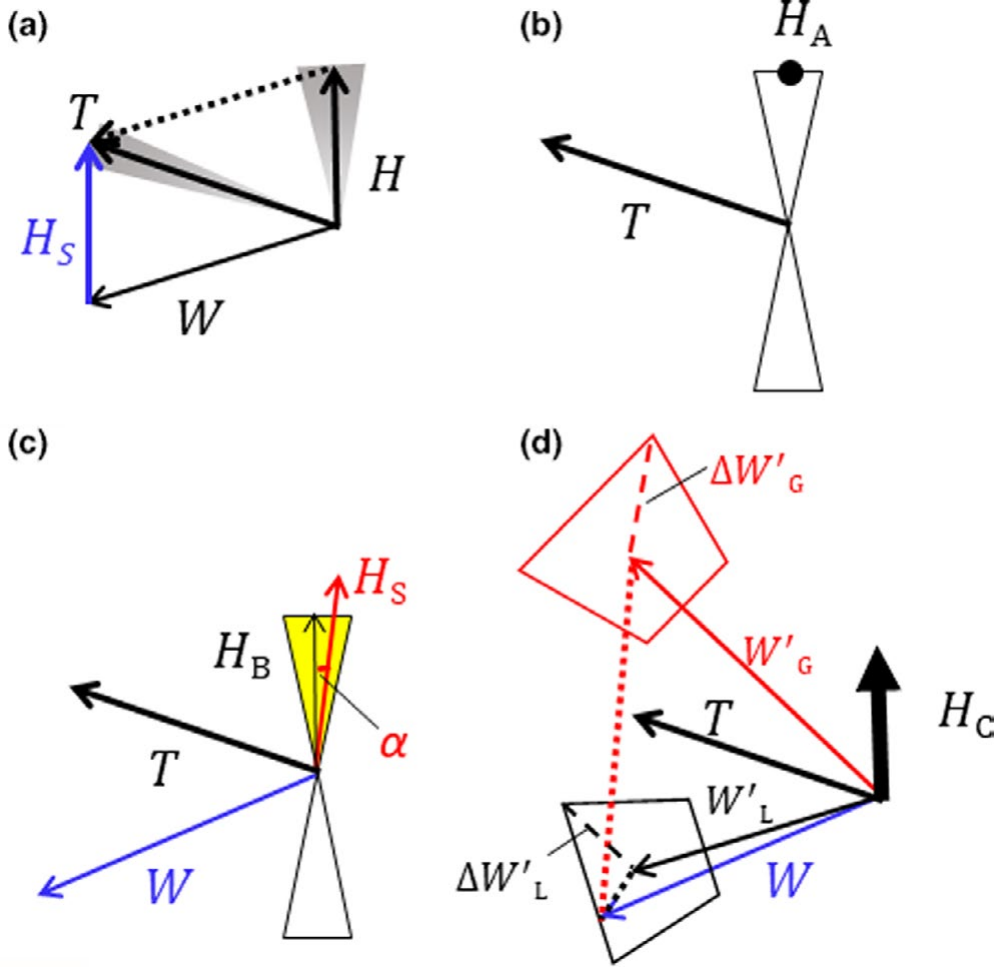
(a) Triangle of track vector ***T***, heading vector ***H*** (and its estimate as subtraction heading vector ***H***_S_), and wind vector ***W*** for insects flying in a wind; gray sectors indicate the spread of ***T*** and ***H*** within a sample. (b) Representation of method A. “Hourglass” triangles represent alignment, the opening angles indicating the circular standard deviation for the unit sample. The black dot indicates the heading direction ***H*_A_** selected by method A_u _(c) Representation of method B. ***H***_S_ is the subtraction heading direction, and yellow fill shows the heading ***H***_B_ selected by method B. *α*, the angle between ***H***_S_ and the closer heading direction, is the subject of the method B quality‐control test. For rejected units, both hourglass triangles are open. (d) Representation of method C. ***W***′_G_ and ***W***′_L_ (G, for the greater distance from ***W***′ to ***W*** and L, for the lesser distance) are the two putative winds, the thick arrow is the selected heading direction ***H***_C_ (method C). The putative wind arrows (which are derived by *subtracting* the heading vector from the track) are red if the corresponding heading direction (not shown) is to the left of the track direction and black if it is to the right. The uncertainties in the two putative winds are plotted as red/black boxes. The red/black dashed lines represent the uncertainty lengths Δ***W***′_G_ and Δ***W***′_L_. The vector distances ***D***_L_ and ***D***_G_ of the putative winds from the model wind are shown as dotted lines. The heading direction selected is plotted black or red if reliable, magenta or blue if tentative, yellow if unresolved according to Equation (3), and open (white) if rejected

To investigate the flight behavior of migrating insects and their response to the wind, it is essential to know their orientations; this requires that the head/tail ambiguity be resolved. We present here two methods of doing this that rely on the vector triangle of velocities that applies to animals moving in environmental flows (Chapman et al., 2008). The heading vector ***H***, incorporating the insect's heading direction and speed relative to the air (the *airspeed*), is the vectorial difference of the track vector (***T***) and the wind vector (***W***; the speed and direction of the wind relative to the ground; Figure 1a). Two possible heading directions are derived from the body alignment measured with the radar. The airspeed is not measured, but some approximate values are available in the literature.

We also consider a simpler method for resolving the ambiguity, one that relies on radar data alone: The heading direction that is closest to the track direction is selected. In some recent studies, radar‐measured insect speeds were 3–6 m s^‒1^ faster than wind speeds from an atmospheric model (Chapman et al., 2008, 2010), and use of this method is then supported. Earlier work combining scanning entomological radar observations of insect migrations with pilot‐balloon ascents also never detected overall backward displacement movement (Riley & Reynolds, 1986). However, with our observations (see below), selecting the heading closest to the track direction sometimes produces implausible variations with time or height, and track speeds are only slightly greater than wind speeds on average (with some negative differences). Thus, an alternative method of heading selection appears necessary.

Winds experienced by insects flying near the surface are relatively easily measured (Riley & Osborne, 2001; Srygley, 2001), but at altitude, in the absence of a co‐located wind profiler or frequent pilot‐balloon observations, it is necessary to rely on values derived from an atmospheric model driven by routine meteorological observations. A model provides a near‐continuous series of wind values and combining these with the similarly continuous observations from a radar allows stronger inferences about orientation behavior, especially its continuity and variation, to be drawn—as the analyses presented here will demonstrate. However, model winds are sometimes incorrect, radar measurements have limited precision, and there is a degree of uncertainty about the insects' airspeeds. Therefore, an ambiguity‐resolution method based on the triangle of velocities must incorporate recognition of uncertainty in the various input quantities and include some form of “quality control” to eliminate occasions when the wind estimates are incompatible with the radar observations.

In this paper, we present our two methods and assess their effectiveness, and that of the simpler method, by applying them to radar observations of migrations of Australian plague locusts (*Chortoicetes terminifera*) obtained with an insect monitoring radar (IMR; Drake, Wang, & Harman, 2002) at Bourke, New South Wales, Australia.

## 2 MATERIALS

### 2.1 Circular statistics

Circular statistics have been used to calculate mean values of directional quantities and two measures of variance, the mean resultant vector length (*R*), and the circular standard deviation (Berens, 2009). *R* ranges from 0 to 1, with larger values of *R* indicating greater deviations of the population from a uniform distribution, that is, more clustering about the mean. The Rayleigh test (Fisher, 1995) was used to test the uniformity of angular distributions, at a significance level of *p* < 0.05.

### 2.2 Radar observations

The radar observation data are from an IMR at Bourke airport (30.0392°S, 145.952°E, 107 m above sea level) and were acquired from September 2010 to April 2011: This period comprises spring (September–November), summer (December–February), and autumn (March–May) of a single insect‐flight year. Australian plague locusts, which were relatively plentiful over this year, were identified from the characteristics of their radar returns (Drake & Wang, 2013; Hao, 2016). The radar observations were recorded every night in three periods of ∼8 min during each hour from 18:00 hr to 06:00 hr Australian Eastern Standard Time (AEST = UTC+10 hr; all times in this paper are AEST). Radar echoes were recorded from heights between 175 and 1,300 m and collated into “units” 150 m deep and 1 hr in duration. For a unit to be included in the analysis, it had to contain at least 12 targets identified as *C. terminifera*. In each unit, outliers of the track speed and the track direction were discarded; the acceptable ranges were two standard deviations from the mean for speed and either two circular standard deviations or 81° (the maximum possible value for a circular standard deviation) from the angular mean for direction. Units were also required to exhibit significant nonuniformity (Rayleigh test, *p* < 0.05) in both track and alignment directions. Only nights of “strong migration”—those with more than 1,000 locust targets and with 25 or more included units—were used in the main analysis.

### 2.3 Wind data

The air pollution model (TAPM; Hurley, 2008) was used to estimate the wind vector ***W*** at the heights the insects were flying. TAPM is designed for high‐resolution weather simulation and has been tested in a wide range of locations, both in Australia and overseas (Hurley, Edwards, & Luhar, 2008). TAPM simulation of boundary‐layer winds has been verified by comparing its outputs with winds measured by sodar and electromagnetic wind profilers at Wagga Wagga, NSW (Hao, 2016; Taylor, Zawar‐Reza, Low, & Aryal, 2005). The results showed that the root‐mean‐square errors are smaller than Australian plague locust airspeeds (approximately 4 m/s; Clark, 1969; Hao, Drake, Sidhu, & Taylor, 2017), so should not dominate our vector‐triangle calculations. For consistency with insect track and heading directions, wind directions in this paper are stated as the direction the wind was traveling *toward* (rather than the direction it was coming *from*, as is usual in meteorology).

## 3 HEADING‐SELECTION METHODS

### 3.1 Method using only radar data

As discussed above, the true heading direction of the insect may be either the alignment value provided by the IMR, or 180° from that value. The simplest method to determine the orientation (method A hereafter) would be to select the heading direction closer to the track direction. This method is considered applicable only to strong‐flying insects and implicitly assumes that these do not head into winds stronger than their airspeed and thus do not exhibit overall backward displacement. (We will refer to such movements as *tailfirst*, and this is to be understood as including the full 180° sector of orientations for which the along‐track component of the heading vector is negative.) Two variants of method A appear possible: Either the “closest” criterion is applied to each echo in a unit individually, and a distribution of heading directions is then obtained (method A_i_) or mean track and alignment directions are calculated for all the echoes in the unit, and the criterion is applied once to these means (method A_u_, Figure 1b).

**Figure 2:**
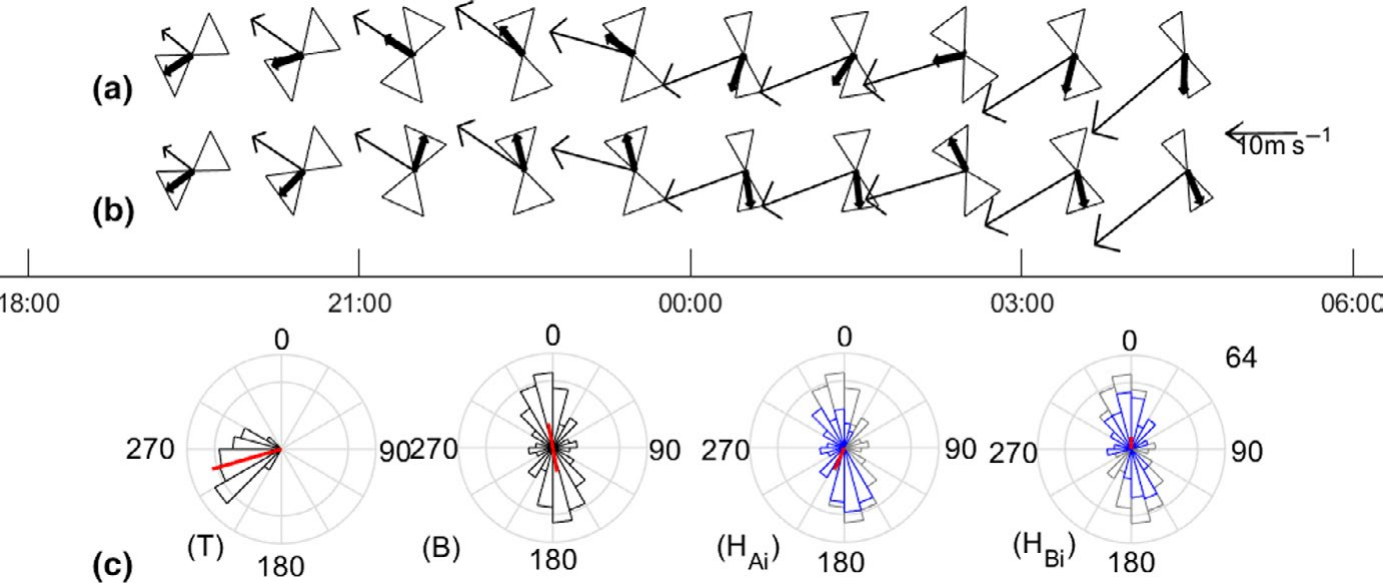
Radar observations of body alignments and track directions of *Chortoicetes terminifera* targets on 6/7 November 2010 for the height interval 475–625 m, at Bourke. Thin arrows represent mean tracks (speed scale at right), hourglasses represent alignmnent distributions, and thick arrows represent means of the selected heading directions; see also Figure 1b. Heading directions are selected by (a) method A_i _and (b) method A_u_. (c) Distributions, for the 00.00–01.00‐hr unit and the same height interval, of track directions (T), body alignment (B), heading directions selected by method A_i _(H_Ai_, blue, with the alignments in gray) and by applying method B to individuals (H_Bi_, blue). Outer ring represents 30 units and inner ring 15 units. Red lines show mean directions, with lengths indicating *R* (radial scale linear, range 0–1, for these)

Method A confines the inferred heading directions to within a 180°‐wide sector around the track directions, and if the assumption of downwind flight is incorrect, this will produce spurious results. An example (from the locust dataset of this paper) where this appears to have occurred is shown in Figure 2. While the alignment varied gradually through the night, the heading direction inferred from method A reversed suddenly at around midnight and “flipped” back‐and‐forth between 02.00 and 03.00. These erratic changes arise because the track directions are almost perpendicular to the alignments, and a small change in track direction flips the selection from one alignment direction to the other. When method A_i_ is employed (Figure 2a), the mean heading direction for five of the units lies outside the range of the radar observations of body alignments. The problem is that when the tracks and alignments are almost perpendicular (but show variation between individuals), the procedure will assign some echoes to one alignment direction and some to the opposite one. This is evident between 00:00 and 01:00, when the distribution of heading directions inferred by method A_i_ is split with maxima toward both the NNW and the SSE (Figure 2c, H_Ai_), even though there is a unimodal track direction distribution (Figure 2c, T) and a concentrated distribution of body alignments (Figure 2c,b). The split distribution leads to a mean heading direction that is inconsistent with the alignment observations. When the selection is based on units (Figure 2b), rather than individual targets, the split heading distributions do not arise, but the results are still problematic as flipping of the inferred heading directions still occurs (and in fact is more frequent). The failure of both variants of method A in this example is due to the almost perpendicular track and alignment directions a phenomenon that occurs commonly in Australian locust migrations (Drake, 1983; Rennie, 2013).

**Figure 3:**
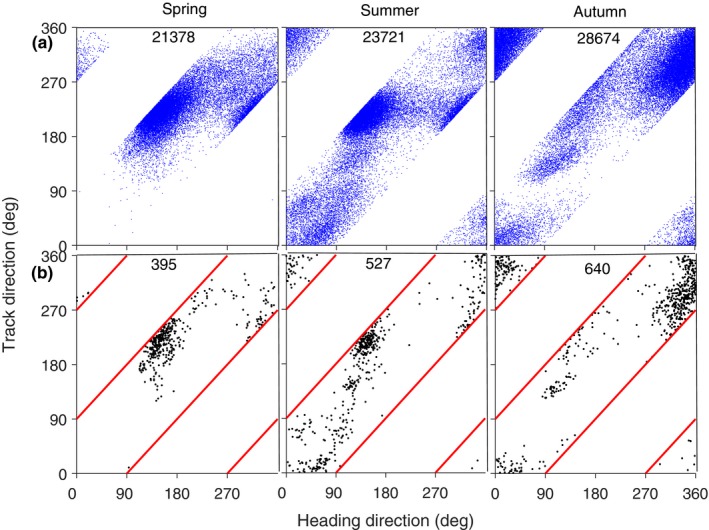
Heading direction selected by method A in relation to track direction, for locusts at Bourke in spring, summer and autumn 2010–2011. (a) Selection on individuals, (b) on units. The number on each panel in (a) is the number of insects included on the plot and in (b) is the number of units. The directions 0°, 90°, 180° and 270° represent N, E, S and W

When method A is applied to the strong migration nights of 2010–2011, the track directions and heading directions are seen to be positively related in all three seasons (Figure 3), but this is an inevitable consequence of not allowing tail‐first movement. The diagonal white (i.e., unpopulated) regions are where tail‐first movements would appear (Figure 3a). The sharp edges to the distribution of points adjacent to these diagonals are clearly artifactual and suggest the true distribution extends into these regions, indicating that some tail‐first movement did occur. When the procedure uses units rather than individuals (method A_u _rather than method A_i_), the sharp edges are less apparent (Figure 3b); however, they are evident in a larger dataset (with 7 years of radar observations; see Fig. 7.5a in Hao, 2016). This longer study provides additional indications of tail‐first movement as locust track speeds were found to exceed the corresponding wind speeds by only 1.2 m/s on average (both mean and median), which is much less than the 4 m/s airspeed of *C. terminifera* or the 3–6 m/s speed difference seen by Chapman et al. (2010), and there was a proportion of negative differences (Hao, 2016, Fig. 7.1). Thus, while method A may give reasonable results in some circumstances, it does not appear reliable for use with Australian locusts, or indeed for any migration in which alignments are at large angles to tracks—that is, in which there is strong drift.

From this examination of the case illustrated in Figure 2, some fundamental expectations about the output from a valid heading‐selection procedure can be identified (see Discussion). These expectations have guided our development of the two new methods proposed here and underlie the analyses undertaken to validate them.

### 3.2 Methods incorporating wind data

Our two methods are fundamentally equivalent, drawing on the triangle of velocities, but the procedures employed differ considerably. As the atmospheric model provides winds at hourly intervals, there is only one wind value for each unit and it seems sensible to relate this to single unit‐average values of insect tracks and alignments, doing so also avoids the generation of split distributions. With these methods, the inferred heading directions can range over 360°, that is, they may determine that the insects are moving tailfirst. In some instances, however, a vector triangle that is consistent with the radar observations of insect alignment, and that has reasonable values for insect airspeeds, cannot be formed, and there is then no basis for inferring a heading direction. Our procedures therefore incorporate quality control, so that unreliable results are identified and eliminated from samples. This of course reduces the size of the sample and could possibly introduce bias. The tests allow a wide 1–7 m/s range for the airspeed, to account for variance in, and a degree of uncertainty about, this 4 m s^−1^ value.

As mentioned, an insect's movement over the ground, expressed by the track vector ***T***, is the vector sum of the heading vector ***H*** and the wind vector ***W*** (Figure 1a). We assume that the direction of the vectorial difference between ***T*** and ***W***, that is, of the insect's “heading vector by subtraction” (***H***_S_, blue arrow), is aligned with the insects' body axis, that is, that the insects fly without sideslip. We can estimate ***H***_S_ from radar measurements of ***T*** and values of ***W*** from an atmospheric model and use it to resolve the head/tail ambiguity by choosing the alignment direction that is closer to the ***H***_S_ direction (***H***_B_ in Figure 1c). This constitutes our first new method (method B hereafter). We apply method B only to units, because if applied to individuals, split distributions (Figure 2c, plot H_Bi_) arise just as in method A_i_. Quality control is implemented through tests that the ***H***_S_ speed is within the range 1–7 m/s, and the difference between the direction of ***H***_S_ and the closer of the two mean heading directions for the unit (*α* in Figure 1c) does not exceed 2*σ*, where

(1) $$\sigma =\sqrt{\sigma_{T}^{2}+\sigma_{H}^{2}}$$

where *σ_T_*and *σ_H_* are the circular standard deviations for the unit of, respectively, the track direction and the alignment.

**Figure 4:**
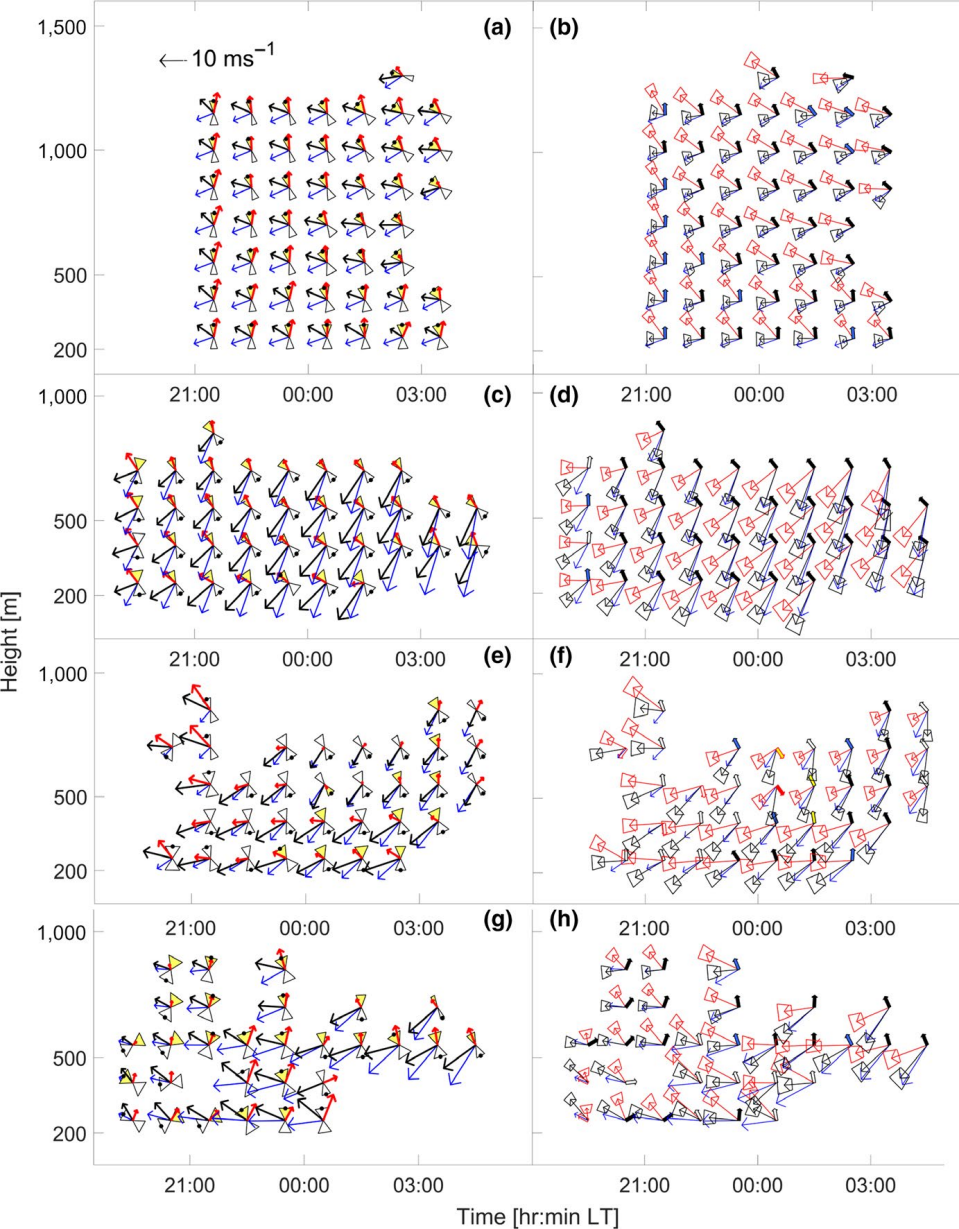
Method B (a, c, e, g) and method C (b, d, f, h) analyses for four case studies of *Chortoicetes terminifera* migration at Bourke. (a, b) 13/14 March 2011 (case 1), (c, d) 12/13 October 2010 (case 2), (e, f) 11/12 November 2010 (case 3), and (g, h) 6/7 November 2010 (case 4). See Figure 1(b,c) for key to symbols. Note speed scale at top left, which is same for all vectors; the length of the hourglass triangles indicate an airspeed of 4 m/s

The second new method (method C hereafter) approaches the vector‐triangle test from the opposite direction. For each unit, two “putative wind” vectors ***W***′ are calculated from the mean track vector by using the two possible mean heading directions and the 4 m/s airspeed (Figure 1d). Uncertainties in the putative winds are calculated from the spread of heading directions, track directions, and track speeds (plus an extra ±10% of track speed to allow for possible systematic error in this quantity), and a 25% (i.e., 1 m/s) uncertainty in the airspeed. The putative wind that is closer to the simulation wind is identified (from the lengths of the difference vectors ***D***_L_ and ***D***_G_, Figure 1d), and the heading direction that produced this putative wind is selected. In this method, quality control is applied through the ratio

(2) $$R_{w\prime }=\frac{\left|D_{L}\right|}{\Delta W_{L}^{\prime }},$$

where |***D***_L_| is the length of the difference vector which is lesser and Δ***W***′_L_ is the magnitude of the uncertainty in the selected putative wind vector, which is estimated as the distance to a corner of the uncertainty trapezium (Figure 1d; this being the quadrature sum of the lateral and longitudinal uncertainties). If *R_W_*_′_ ≤ 2, then that putative wind and the selected heading direction are considered to be reliable; if 2 < *R_W_*_′_ ≤ 3, the selection will be considered as tentative (and will be plotted in different colors, see e.g. Figure 4f); and if *R_W_*_′_ > 3, the unit fails quality control. For some units, both putative winds may have passing *R_W_*_′_ values, and to resolve the selection, we then require that one is clearly in better agreement with the model wind than the other. This test is implemented as

(3) $$R_{w\prime }<1.5\frac{\left|D_{G}\right|}{\Delta W_{G}^{\prime }},$$

that is, the rejected distance must be at least 50% greater than the selected one.

## 4 RESULTS

Analysis of the 2010–2011 radar observations revealed that there were 46 nights of strong migration (as defined previously). On one of these nights, track speeds were very low and orientations changed apparently randomly before midnight, so this case has been excluded from the sample. From the remaining nights, we have selected four cases to represent different scenarios and for each of these, we show how the simpler method and the two new methods performed. We also present a summary of their performances over all 45 nights.

### 4.1 Case studies

For the night of 13/14 March 2011 (case 1), all three methods produce similar results (Figure 4a,b). The wind maintained a southwest direction through time and height, while the locust track directions were toward the northwest all night at lower heights but shifted to westward later at higher levels. Body alignments are approximately north/south early in the night and low down, and northwest/southeast later and higher up. Both body alignments and track directions were highly concentrated throughout the night and exhibited only gradual variations. According to all three methods, the locusts were heading north at 21:00 and gradually changed to a northwestward heading (Figure 4a,b). All quality‐control tests (methods 2 and 3) passed.

For 12/13 October 2010 (case 2), the new methods select headings that disagree with those obtained by method A (Figure 4c,d). The wind was blowing to the southwest at ~10 m/s from 19:00, gradually increasing in speed to ~15 m/s after midnight. Alignments are north/south initially, but then northwest/southeast for most of the night. Tracks are toward the southwest, and so are at a large angle to the body alignments. When selected by the track direction (method A), all but one of the headings are to the southeast. (the exception, at 23 hr, represents a flip followed immediately by a second reversal.) In contrast, method B consistently indicates that the locusts were flying toward the northwest, and the computed airspeeds are reasonable (Figure 4c). The same result is obtained with method C, as the putative winds derived from the track vectors and heading directions to the north or northwest are quite closely aligned with the model winds while their counterparts with southward or southeastward headings are at much larger angles to them (magenta arrows, Figure 4d). There are no flips, and after the first hour, the quality‐control requirements are met for all units. Both new methods show that the locusts were heading toward the northwest but were being blown sideways and slightly backward by the strong winds, so that they were undertaking tail‐first movement.

Case 3, the night of 11/12 November 2010, provides an example of simulated winds that cannot be reconciled with the track and body alignment information from the radar. During the early part of the night, the wind simulations appear to have been poor as the quality‐control tests fail; however, after midnight, the model winds become compatible with the radar data and the two new methods produce mostly consistent heading selections (Figure 4e,f). A similar scenario, of TAPM outputs being wrong initially but improving later, occurs also on other nights. As in case 2, the headings selected by method A often differ from those obtained with the new methods. The method‐1 headings flip across both time and height early in the night, whereas the new methods eliminate these units from the sample. However, on this occasion, method C also produces a flip, at 23.00.

**Figure 5:**
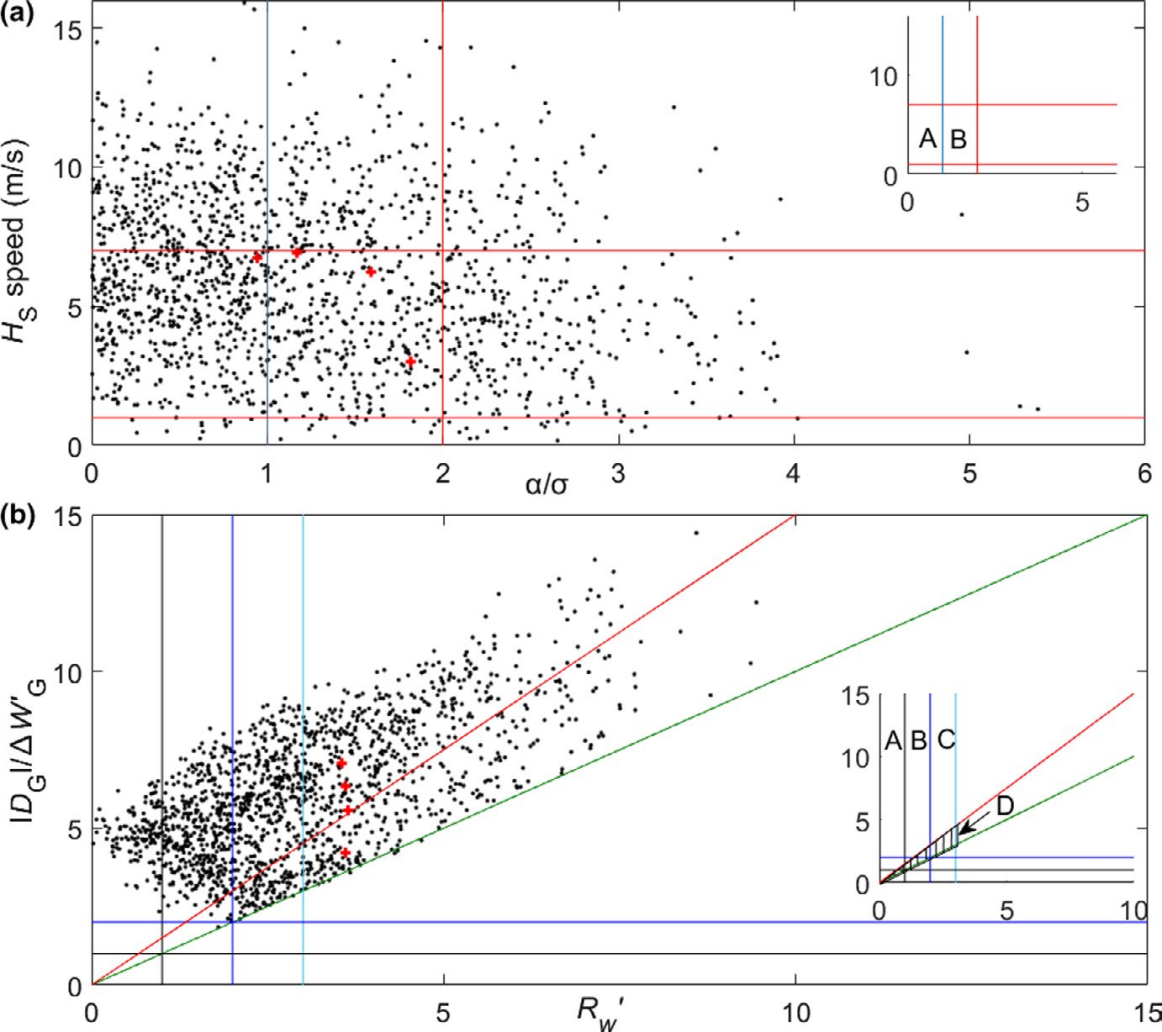
(a) Scatter plot for method B, showing ***H***_S_ speed against the angle *α* (see Figure 1b), with the angle scaled by the uncertainty on it (Equation 1). (b) Scatter plot for method C, of the length of the difference vector ***D***_L_ for the closer putative wind (see Figure 1d) against the corresponding length ***D***_G_ for the more distant putative wind, with both lengths scaled by their uncertainties. The red crosses indicate the units in case 4 that were accepted by method B but rejected by method C. The insets identify regions referred to in the text

For the night of 6/7 November 2010 (case 4), both new methods indicate that the simulated winds are inaccurate at lower levels during much of the night (Figure 4g,h). Method A is not reliable, as shown by its heading selections flipping through both time and height. Method B rejects 6 units and method C rejects these and 4 more; but they give consistent headings for those remaining. The units rejected by both new methods are largely at lower heights, especially between 325 and 475 m, a zone where a low‐level jet wind (see Discussion) is quite likely to have developed. The four units for which the two new methods give different rejection statuses (red crosses in Figure 5) are mostly in situations close to the boundary between acceptance and rejection (see next section).

### 4.2 Effect of quality control

For our 1‐year sample, 53.7% of units were rejected by quality control when using method B, and 49.2% with method C. The manner in which these rejections occur is indicated in the scatter plots of Figure 5. For method B, the ***H***_S_ vector requirements (see Section 3.23.2) are satisfied only in regions A and B of Figure 5a. For method C, units are selected when one putative wind, and its associated heading selection, is considered very reliable (regions A and B of Figure 5b) or tentative (region C), and its counterpart is not (i.e., excluding region D). The nonselected putative winds are almost always clearly incompatible with the simulated winds (i.e., their *R_W_*_′_ is >2), so the number of units rejected via Equation 3 (i.e., that fall within region D) is quite small (6.2%), with most in the tentative category. In Figure 5b, the data appear as a band with restricted width; this is due to the difference between the two putative winds being limited by the assumed locust airspeed of 4 m/s (which falls to ~3 m/s with vector averaging over the various individual heading directions).

### 4.3 Full‐year dataset

Some statistics for performance of the methods over the 45 nights of strong migration are presented in Table 1. Model winds and radar observations were incompatible for ~45% of units, rendering ~40% of nights unusable for analyses of locust migratory behavior. Rather more units were found to be compatible by method C than by method B, suggesting the method C quality control may be less stringent, or perhaps that this method works better. When both methods B and C give a result, these differ for only ~1% of units. There was agreement between all three methods for ~20% of units, and disagreement between the simpler method and two new methods for a similar proportion; the equivalent proportions for nights were similar. These two categories correspond to forward and tail‐first movement, and in this dataset, it appears that these occur with approximately equal frequency.

**Table 1:** Numbers of units and nights for different scenarios during 45 nights of strong locust migration between September 2010 and May 2011

Scenarios	Number	%
Units		
Results from method B	728	46.6
Results from method C	795	50.9
No results from one or both of methods B and C	904	57.9
No results from both method B and method C	697	44.6
Three methods agree	354	22.7
Only new methods agree (tail‐first movements)	295	18.9
Two new methods disagree	9	0.5
Total	1,562	–
Nights		
Winds are partially or fully wrong (methods B and C both fail for ≥50% units; example case 3)	18	40.0
Three methods agree (indicated by ≥70% units agree; example case 1)	9	20.0
Only new methods agree (indicated by ≥70% units agree; tail‐first movements; example case 2)	11	24.4
All methods agree for part of the night and the other parts have some tail‐first movements (example case 4)	7	15.6
Total	45	–

**Figure 6:**
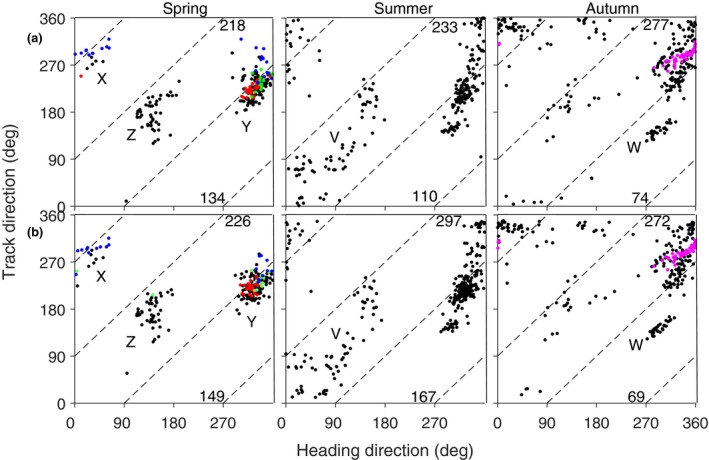
Heading direction in relation to track direction for locusts at Bourke in spring, summer, and autumn 2010–2011 from (a) method B and (b) method C. The magenta dots indicate the units from case 1, red case 2, green case 3, and blue case 4. The letters X, Y, Z, V, W identify the adjacent clusters of dots. The numbers in each panel are the number of units included on the plot (top) and the number showing tail‐first movements (bottom)

Scatter diagrams of headings versus track directions for the units retained by each of the new methods are shown in Figure 6, with the units from the four case studies distinguished by color. Unlike Figure 3, these scatter plots do not contain excluded regions (diagonal empty bands), as all heading directions are possible. The boundaries of the method‐1 exclusion regions are shown, however, and it is evident that there are clusters of points that straddle them, that is, that extend into these regions. In fact, the two new methods show that locusts often make tail‐first movements. By method B, 61.5% in spring, 47.2% in summer, and 26.7% in autumn are tailfirst, and by method C, the corresponding proportions are 65.9%, 56.2%, and 25.3%.

Overall, the heading directions selected by the two new methods are very similar (compare Figure 6a,b) but differ significantly from those selected by method A (Figure 3b). The track direction is related to heading to some degree, with this relationship being slightly different across seasons. The summer pattern appears to be a mixture of the spring and autumn patterns. During spring, three clusters of points are revealed by both new methods, although two of these (X and Y in Figure 6) could be combined as the headings wrap around from 360° to 0°. This cluster, with headings to the NW and N, indicates a preferred direction which is not evident in Figure 3b (spring) where many of the headings are incorrectly assigned. In autumn, this cluster is still present but the track directions have shifted from SW to WSW and NNW, so tail‐first movements are not so frequent; however, there is now also a smaller cluster (W) with heading directions still toward the NW but tracks to the SE. The third cluster in spring (Z) has heading directions toward the SE and tracks to the SE and S; it apparently represents a different flight behavior, perhaps in response to different winds, or possibly a different population group. This cluster is also present in summer and (though weaker) in autumn. In summer, but not the other two seasons, there is a diffuse cluster (V) with headings and tracks to the N and E; this exhibits no tail‐first movement and in consequence is also evident in Figure 3b.

## 5 DISCUSSION

In this paper, the simpler method of using track direction to select heading direction from the ambiguous radar observations of alignment and two new methods that also draw on wind information have been examined. For the 1‐year study period and the Australian plague locust migrations considered here, the headings selected by the simpler method and the two new methods differ for around half of the time‐height units for which comparisons can be made. Moreover, some of the headings selected by the simpler method are clearly incorrect. This is because that method incorporates artificial limits, which lead to flips in the heading direction (Figure 2b) and splits (with artifactual sharp edges) in obvious clusters and bands in track‐heading scatter plots (Figure 3). The two new methods present a significantly different, and presumably more accurate, picture of the relation of heading to track. However, use of these methods leads to quite a large proportion of the sample being lost because the model wind is quite often incompatible with the radar observations. Note that the track and alignment observations are still valid: The rejections arise from poor wind estimates. This frequent occurrence is not unexpected given the difficulty of predicting the diurnal variation of low‐level winds over land areas, and especially the development of a low‐level jet (Hao, 2016, pp 47–69)—a feature that often coincides in both height and time with insect migrations. These rejections, and the associated loss of sample data, would presumably be largely eliminated though installation of a boundary‐layer wind‐profiling system at the radar observation site.

Our results show that the two new methods, which differ only in how they implement the same fundamental test, produce similar results and rejection rates. There thus appears to be little reason to prefer one over the other. Method B is more intuitive and readily interpretable, method C more akin to a formal statistical test. Methods A and B can be applied either to individual insect targets or to samples (such as our one unit), but we have shown that application to individuals leads to artifactual splits and should not be implemented. Method C can only be based on samples as it requires estimates of uncertainties, which are derived from sample statistics.

From the analyses presented here, and building on the earlier examination of Figure 2, five principles for assessing heading‐selection methods can be identified. (1) The selected heading directions must be compatible with the measured alignment directions. (2) Insect airspeeds estimated, assumed, or implied in the procedure must be consistent with known or reasonable values for the species being observed. (3) If track and alignment distributions are unimodal, then the distribution of selected heading distributions should also be unimodal. (4) If mean tracks and alignments, and model winds, vary smoothly with height and time, then mean heading directions should usually also vary smoothly. (5) If track and heading combinations form clusters, indicating similar behavioral responses, these should not be split or exhibit sharp edges. Principles (1) and (2) form the basis of the quality‐control tests incorporated into our methods, and principle (3) leads to our rejection of individual‐based heading selection. Principles (4) and (5) provide a basis for validating methods, though we note that some degree of expert judgment will remain necessary to distinguish artifactual effects from real, and perhaps unforeseen, migrant responses to changing winds and to other varying environmental cues.

## CONFLICT OF INTEREST

None declared.

## AUTHOR CONTRIBUTIONS

All authors contributed to conception of the study, design of the procedures, and analysis and interpretation of the data. Zhenhua led the writing of the manuscript. All authors critically reviewed drafts, contributed revisions, and gave final approval for publication.

## Data Availability

Radar data and model winds for the 45 nights are available on the Dryad data repository at http://dx.doi.org/10.5061/dryad.b28t0q7.
